# Precursor Lesions of High-Grade Serous Ovarian Carcinoma: Morphological and Molecular Characteristics

**DOI:** 10.1155/2010/126295

**Published:** 2010-04-27

**Authors:** Amy L. Gross, Robert J. Kurman, Russell Vang, Ie-Ming Shih, Kala Visvanathan

**Affiliations:** ^1^Department of Epidemiology, Johns Hopkins Bloomberg School of Public Health, Baltimore, MD 21205, USA; ^2^Division of Gynecologic Pathology, Department of Pathology, Johns Hopkins University School of Medicine, Baltimore, MD 21287, USA; ^3^Department of Medical Oncology, Johns Hopkins University School of Medicine, Baltimore, MD 21287, USA

## Abstract

The lack of proven screening tools for early detection and the high mortality of ovarian serous carcinoma (OSC), particularly high grade, have focused attention on identifying putative precursor lesions with distinct morphological and molecular characteristics. The finding of occult invasive and intraepithelial fallopian tube carcinomas in prophylactically removed specimens from asymptomatic high-risk BRCA 1/2-mutation carriers supports the notion of an origin for OSC in the fallopian tube. The intraepithelial carcinomas have been referred to as serous intraepithelial carcinomas (STICs) but our own findings (unpublished data) and recent reports have drawn attention to a spectrum of changes that fall short of STICs that we have designated serous tubal intraepithelial lesions (STILs).

## 1. Introduction

Ovarian cancer is the most lethal gynecologic cancer, responsible for over 13,000 deaths in the US in 2009 (http://www.cancer.org). The majority of these cancers are detected at an advanced stage, after they have spread to the peritoneal surfaces; the 5-year survival rate for women diagnosed at stage III-IV is only 28% (SEER Cancer Statistics Review, 1975–2004, National Cancer Institute http://seer.cancer.gov/csr/1975_2006/). A lack of effective screening tools for early detection of ovarian cancer in high-risk and general populations has led to increased interest in the identification of precursor lesions defined by both morphological and molecular changes that could be the target of not only early detection but prevention efforts.

## 2. Molecular Characteristics of Ovarian Cancer

Ovarian cancer is heterogeneous, like other cancers, comprising a collection of subtypes with different histologic and molecular characteristics that in turn inform prognosis [1]. Accumulating evidence suggests that there are two general pathways in the molecular pathogenesis of what is known as ovarian cancer [2, 3]. The first (Type I) pathway leads to borderline tumors, which can develop into low-grade serous, mucinous, endometrioid, and clear cell carcinomas. These are for the most part low-grade tumors that are characterized by a high frequency of mutations of *KRAS*, *BRAF, ERBB2, CTNNB1* (the gene encoding beta catenin), and *PIK3CA*, low proliferation, and a 5-year survival of approximately 55% [2]. A stepwise model of progression from cystadenomas to low-grade carcinomas has been proposed for these neoplasms. 

 In contrast to the Type I tumors, the Type II tumors are high-grade and highly aggressive, spreading rapidly throughout the pelvis. Type II tumors include high-grade serous carcinoma, malignant mixed mesodermal tumors, and undifferentiated carcinomas. They are characterized by a high frequency of mutations in *TP53*, a tumor suppressor gene, and a high proliferative index. It is estimated that 60% of sporadic ovarian carcinomas and the majority of those diagnosed in BRCA1 mutation carriers are of the high-grade serous type [4, 5]. Preliminary data suggests that these TP53 mutations may develop early in the carcinogenic process. If in fact this is confirmed, new approaches to early detection and prevention can be developed.

## 3. Identifying the Cell of Origin

It has conventionally been assumed that ovarian cancers arise from the ovarian surface epithelium (OSE), which is viewed as a modified type of mesothelium similar to that which lines the peritoneal cavity, by a process of invagination leading to the development of cortical inclusion cysts (CICs). It has been argued that changes in the microenvironment of the ovarian stroma surrounding the CICs leads to müllerian-type differentiation. This step, which is viewed as a metaplastic change, would be necessary to explain the morphologic appearance of ovarian epithelial tumors which have a müllerian-type phenotype. However, others believe it is unlikely that CICs are precursors and have instead proposed that ovarian epithelial tumors develop from müllerian-type epithelium lining paraovarian and paratubal cysts (the so-called secondary müllerian system) [6]. A tubal origin for high-grade serous ovarian cancer is supported by gene expression profiles of OSC that reveal that they are more similar to normal müllerian epithelium than the ovarian surface epithelium [7].

## 4. Prophylactic Salpingo-Oophorectomy in BRCA1 and BRCA2 Mutation Carriers

Prophylactic bilateral salpingo-oophorectomy (BPSO) specimens from high-risk women have proved to be an invaluable resource for research into the origins and precursors for high-grade serous pelvic carcinomas. Women found to have a deleterious germline mutation in the *BRCA1* or *BRCA2* gene are known to be at increased risk for ovarian cancer, with lifetime risk estimates ranging from 40% to 60% [8, 9]. Given the limitations of current options for ovarian surveillance, bilateral prophylactic salpingo-oophorectomy (BPSO) after the completion of child-bearing is the current standard recommendation for these women [10]. This procedure greatly reduces the risk of subsequent development of pelvic serous carcinoma, by 80%–90%, but surprisingly does not eliminate it entirely [11]. Remaining risk is mainly attributed to primary peritoneal serous carcinoma, which is similar to high-grade OSC in terms of presentation and response to treatment [12] and appears to originate from the same cell lineage [11]. The estimated cumulative incidence of peritoneal cancer at 20 years after oophorectomy is 4.3% [13], and so far a survival benefit has been shown in the short term but not long term [14].

## 5. Occult Carcinoma

It is estimated that between 2% and 17% of all BPSO specimens from BRCA1/2 mutation carriers will contain an occult cancer (see Table 1). The range of estimates to some extent is reflective of the comparison groups and the protocols used for sectioning specimens [11, 13, 15–24]. Despite these inconsistencies a majority of early serous cancers found in these specimens are localized to the fallopian tube.

## 6. Serous Tubal Intraepithelial Carcinomas

**Figure 1:**
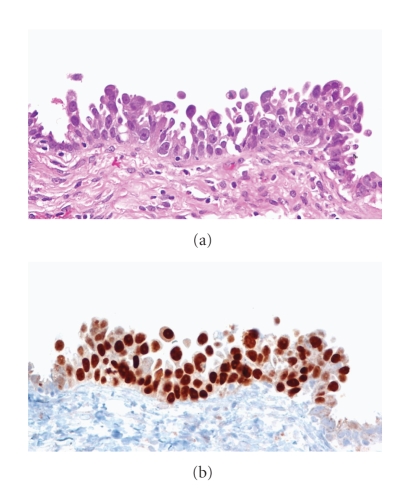
Serous tubal intraepithelial carcinoma (STIC). (a) The morphological features of a small STIC showing papillary architecture with intraepithelial carcinoma cells, many of which are detached and are able to freely disseminate onto ovaries, pelvic, or peritoneal wall. (b) The p53 immunohistochemistry demonstrates that all intraepithelial carcinoma cells are intensely positive for p53 nuclear immunoreactivity. In contrast, the normal-appearing tubal epithelial cells flanking the STIC are negative for p53.

High-grade serous tubal intraepithelial carcinomas (STICs) are noninvasive carcinomas of the fallopian tube that have been found with varying frequency in BPSO specimens (see Table 2). STICs are characterized morphologically by nuclear hyperchromasia and atypia, mitotic figures, and nuclear stratification [26]. Immunohistochemically, they exhibit increased staining for p53 and MIB-1 (Ki-67) [26] (Figure 1). As noted above, many of the occult malignancies detected on thorough sectioning of BPSO specimens were microscopic and restricted to the fallopian tube. Subsequently, a careful and thorough examination of the fallopian tubes from 55 consecutive cases of “serous carcinoma” (pelvic, ovarian, or tubal) revealed that over 70% involved the endosalpinx and approximately half contained STICs [27]. These findings led to the hypothesis that the fallopian tube may be the source of a significant proportion of all pelvic high-grade serous carcinomas. To further confirm the shared origin of these OSC with their coexisting STICs, the authors analyzed p53 mutations in both sites from five cases. Identical mutations were detected in both sites for all cases. An analysis of chromosomal copy number changes by FISH demonstrated similar results in 3/5 cases comparing ovarian serous tumors with synchronous fallopian tube serous carcinoma, providing some additional potential support for a common, monoclonal origin [28]. Further, another study from the same group examined 45 cases of primary peritoneal serous carcinoma and found that 9 out of 26 cases with incomplete tubal sampling and 9 out of 19 cases that underwent complete examination of the tube had STIC [29]. In all of these studies, the majority of STICs were found in the fimbriated end of the tube, adjacent to the ovarian surface.

 In an effort to verify the fimbria as a preferred site for STICs in BPSO specimens, Medeiros et al. [25] investigated 13 BRCA+ BPSOs and 13 controls, who were women who had bilateral salpingo-oophorectomy due to other gynecological diseases. Six of the cases had mutations in BRCA1 and 7 were BRCA2 positive. Cases and controls had similar age ranges and mean ages (50 and 58 years, resp.), and all specimens were entirely submitted for review, sectioned at 2-3 mm intervals. In addition, all fimbriae were extensively sectioned in one of two ways: serial sectioning (7 cases) or by SEE-FIM protocol—Sectioning and Extensively Examining the Fimbriae—in 19 cases. The SEE-FIM protocol was developed to ensure maximal examination of the fimbria [25]. Cases were compared to controls for the rate of detection of early neoplasms, their locations, and expression patterns for p53 and Ki-67. Five cancers were identified, all of which were tubal and from the case group. Four of the five tumors involved the fimbria; four of these five also stained positive for both p53 (>75% nuclei staining positive in a region of 12 cells in length) and Mib-1. The reason for the tendency of these early cancers to be found in the fimbria is not entirely clear, but the authors suggest that it may be due to increased surface area of this site, or potential differences in characteristics of the cells from this region versus more proximal sections of the tube. Their findings support a possible means of spread to the ovary by exfoliation or tubal-ovary adhesions.

## 7. Resemblance of STIC to Serous Endometrial Intraepithelial Carcinoma

Like high-grade OSC, uterine serous carcinomas are aggressive cancers with poor prognosis [30, 31]. Despite comprising only 10%–15% of all endometrial carcinomas, USC causes a disproportionate number of deaths and appears to have a different etiologic pathway than the usual type of endometrial carcinoma (endometrioid adenocarcinoma). In contrast to the endometrioid type, which is typically found to be associated with endometrial hyperplasia and other signs of hyperestrogenism, most uterine serous carcinomas are diagnosed in older, postmenopausal women with atrophic endometrium and no evidence of endometrial hyperplasia. It has therefore been hypothesized that uterine serous carcinoma and its presumptive precursor, serous endometrial intraepithelial carcinoma, may originate in an estrogen-independent manner [32] and are associated with other factors [33]. 

 Serous endometrial intraepithelial carcinoma (SEIC) is characterized by “replacement of endometrial surface epithelium and glands by malignant cells that resemble invasive high-grade endometrial carcinoma” [34]. It has been identified in greater than 90% of uteri containing serous carcinoma [31]. Interestingly, immunostaining for p53 expression in pairs of uterine serous carcinoma with SEIC showed the majority to be p53 positive and all pairs were concordant [34]. Shared p53 mutations have also been described in SEIC and the associated uterine serous carcinoma [35]. Uterine serous carcinoma and OSC both exhibit a tendency to spread rapidly throughout the pelvis and multifocal disease is often found at diagnosis. These multiple foci are thought to represent monoclonal disease, unlike endometrioid carcinomas, which have been shown to have multiple sites of origin [36]. The question of site of origin for these multifocal uterine serous carcinoma parallels that of the origin of other pelvic serous carcinomas.

 A recent study attempted to document the frequency of concurrent STIC and SEIC, conjecturing that uterine serous carcinoma with STIC might represent a distinct subset of pelvic serous carcinomas, with as-yet unclear origin. Of 22 uterine serous carcinoma cases examined, the presence of STIC was confirmed in 5 [36]. It was found that the endometrial tumor in all five of these cases was either noninvasive or superficially invasive, and in 2 of the cases identical p53 mutations were identified in both tubal and endometrial lesions, suggesting a common origin, perhaps in the tube, for these cases. The proposal that these uterine tumors may also be of tubal origin is intriguing and certainly deserves further investigation.

## 8. Identification of Precursor Lesions

### 8.1. Serous Tubal Intraepithelial Lesions

#### 8.1.1. Molecular Changes

**Figure 2:**
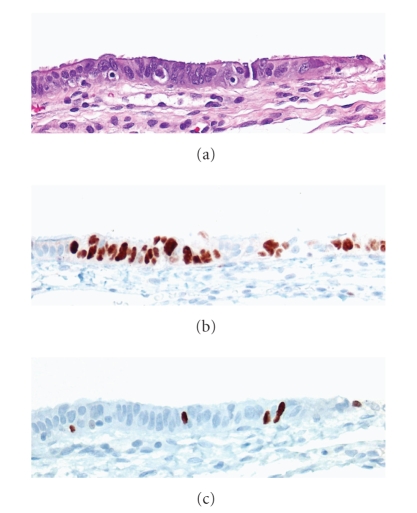
Serous tubal intraepithelial lesion (STIL). (a) The morphological features of an STIL identified in a young cancer-free BRCA mutation carrier. The epithelium shows mild nuclear and architectural atypia but not to the level of a classical STIC. (b) An immunohistochemical stain for p53 shows overexpression in several consecutive secretory cells. (c) An immunohistochemical stain for Ki-67 shows a low proliferation index.

We use the term “serous tubal intraepithelial lesions” (STILs) to describe a spectrum of epithelial changes ranging from normal appearing tubal epithelium, expressing p53, to lesions with increasing degrees of cytologic atypia that fall short of an STIC (Figure 2). Others have reported these changes as tubal intraepithelial lesions in transition (TILT) [37]. Characterization of STILs has become a central focus of several groups, including ours, in order to determine the nature of these lesions and their relationship to STICs, specifically whether these are the earliest steps in the carcinogenic process. A 2007 study [38] sought to examine the relationship of what was designated “p53 signatures,” characterized by high p53 immunostaining, defined as strong nuclear staining obscuring nuclear detail in at least 12 consecutive secretory nuclei in benign-appearing epithelium, and STIC. The fallopian tubes from three groups of women were included in this series: (1) 41 women with BRCA1 or BRCA2 mutations undergoing BPSO, (2) 58 consecutive women undergoing procedures for other gynecologic disease, and (3) 17 women already identified to have STICs, all of which were shown in a previous study to be associated with pelvic serous carcinoma [27]. All fimbriae were sectioned using the SEE-FIM protocol. Sections were examined for prevalence of p53 signatures. Similar percentages of p53 signatures were found in the benign BPSO specimens from BRCA+ women versus group 2 (24% and 33%, resp.) and were most often found in the fimbriated end of the tube. Nine of the 17 STICs (53%) contained at least one p53 signature and multiple p53 signatures were found in tubes containing STICs at twice the frequency found in nonneoplastic tubes. Staining of a subset of tissues containing p53 signature, STIC, or serous carcinoma revealed the colocalization of *γ*-H2AX, an immunohistochemical marker of double-stranded DNA damage, with p53. 

 Another study from the same group compared the prevalence of p53 signatures in fallopian tubes versus ovarian cortical inclusion cysts in BRCA+ women [39]. Cases consisted of consecutive BPSOs performed in BRCA+ women. Controls, taken from a prior study, were a consecutive series of women free of ovarian cancer, having surgery for other gynecologic conditions, who had either tested negative for BRCA mutations or were untested. All tubes and ovaries were submitted entirely, and the SEE-FIM protocol was used for examining the fimbriae. The proportions of specimens that showed at least one p53 signature were similar between cases and controls (38% and 33%, resp.). No CICs were found to contain p53 signatures. Sixty-six percent of the p53 signatures in cases stained positive for *γ*-H2AX; staining for *γ*-H2AX in the p53 signatures of controls was not reported.

#### 8.1.2. Hormonal Changes

One of the prevailing theories of ovarian epithelial carcinogenesis is that of “incessant ovulation” as a potential causative role in these cancers. Ovulation has been suggested to cause genotoxic damage in the ovarian surface epithelium where ovarian cancers have traditionally been thought to originate; the recent findings of colocalization of *γ*-H2AX with p53 staining in STILs and STICs in the fimbria, adjacent to the ovarian surface, is therefore highly intriguing and will be important to explore further. 

 A recent study exploiting genetic microarray technology compared the expression profiles of normal-appearing fallopian tube epithelium of BRCA+ mutation carriers and healthy controls to serous carcinomas [40]. Fallopian tube epithelium samples from the BRCA+ group (but not the normal controls) were found to cluster closely with serous carcinoma samples. In particular, BRCA+ fallopian tube epithelium taken during the luteal phase (versus follicular phase) expressed gene profiles most similar to those of the serous carcinoma tissues. Two differentially expressed genes, SKIL (ski-like), an oncogene, and DAB2, a tumor suppressor, emerged as candidates that have been implicated in the tumorigenic process [41, 42]. SKIL was found to be overexpressed, and DAB2 underexpressed, in serous carcinoma and BRCA+ luteal fallopian tube epithelium, relative to BRCA+ follicular fallopian tube epithelium samples, suggesting that the hormonal milieu during the luteal phase, particularly in BRCA+ carriers, may predispose to a carcinogenic process in the fallopian tube epithelium. The extent to which hormonal changes during the luteal phase represent a transitory phenomenon, and the manner in which the fallopian tube epithelium might retain a more permanent imprint of such cyclical fluctuations, is unclear. 

 Tubal-mucosal morphology is known to be influenced by hormones [15], although these changes have not been precisely quantified. Presumably, the endogenous and exogenous hormonal milieu plays a key role in the molecular alterations of the fallopian tube epithelium, varying with age and menopausal status. Evidence from epidemiological studies provided further supports the role of hormonal exposures in affecting risk of ovarian cancer. It has been wellestablished in epidemiological studies that use of oral contraceptive pills (OCP) protects against the development of ovarian cancer; use for five or more years reduces risk by approximately 50% [43, 44]. Parity has also been shown in numerous studies to be protective, conferring a 30%–60% decreased risk compared to nulliparity [45]. Two theories have been postulated to explain these findings: that ovulation causes repetitive cell damage/repair which over time increases the risk of mutations or that increasing exposure to gonadotropin-releasing hormones (represented by lower OCP use and parity, or increased ovulatory cycles) may be the mechanism by which ovarian cancer risk is increased. But studies attempting to find a correlation between number of ovulatory cycles and accumulation of p53 mutations in ovarian cancers have been contradictory [46–48]. Schildkraut et al. (1997) examined the ovaries from 197 women with invasive ovarian cancer and found that cases overexpressing p53 (53%) were exposed to significantly more ovulatory cycles than those cases that did not overexpress p53. However, a study by Webb et al. examined 234 cases of invasive ovarian cancer and found no associations between p53 overexpression and other factors including years of ovulation, parity, family history of ovarian cancer, or age. One cross-sectional study examined the association between factors including parity, history of breast cancer, prior chemotherapy, smoking history, tamoxifen use, age at first birth, age at time of oophorectomy, BMI, age at menarche, and oral contraceptive use (never versus ever) obtained from medical record review and the presence of p53 signatures in the tubes of 75 BRCA+ mutation carriers [49]. Thirty eight percent of the tubes showed at least one p53 signature, and parity was significantly associated with the presence of p53 signatures. Due to the cross-sectional nature of the study temporality could not be assessed.

#### 8.1.3. Cortical Inclusion Cysts

Several studies comparing the prevalence of CICs in “high-risk” prophylactically removed ovaries versus normal-risk ovaries have reported inconsistent findings [50]. One study comparing morphology of ovarian surface epithelium of 64 women with a family history of ovarian cancer with 30 women without a family history found significantly more CICs in the high-risk group [51], while others have found no significant difference in the frequency of CICs. CICs also appear to increase with age in both high-risk and normal-risk women, and this needs to be taken into consideration when evaluating its significance as a precursor lesion [50]. Barakat et al. [52] examined ovarian tissues from 18 BRCA1 mutation carriers and 20 age-matched controls and found no significant differences in frequency of inclusion cysts or other morphological alterations. Immunohistochemical staining for p53, ERBB-2, and Ki67 of the CICs also showed no difference in expression between cases and controls. In a cross-sectional study examining the relationship between demographic variables and the presence of CICs in 74 bilateral prophylactic salpingo-oophorectomies (BPSOs) from BRCA1/2 mutation carriers, Folkins et al. [53] found women with ≥7 versus <7 CICs per ovary pair had significantly older age at childbirth and menopause, older age at surgery, and higher body mass index (BMI). Limitations of this study include the small sample size, which prevented the analyses from being adjusted for confounders, such as age, and the retrospective collection of information on risk factors from medical records*.*

#### 8.1.4. Future Directions

Large, well-powered studies with uniform sectioning methodology are needed to better define the prevalence of STICs and STILs in specimens from high-risk women and the general population, and to determine the incidence of subsequent peritoneal cancer. Further prospective data is needed to assess interactions between exposures and these lesions. Animal experiments could assist in validating the temporality of these precursor lesions to the development of ovarian serous carcinoma, as this type of study would not be feasible in clinical studies. The genetic changes accompanying and preceding these lesions may then be able to be targeted in prevention studies. In addition, a validated classification of STIL would help both internal validity and generalizability of studies.

## Figures and Tables

**Table 1:** Prevalence of occult carcinoma in bilateral prophylactic salpingo-oophorectomy specimens.

Author	Study design	Study population	Sectioning protocol	Findings
Colgan et al. 2001 [15]	Cross-sectional	60 BPSO specimens from women (mean age 48.4) with high likelihood of being BRCA mutation carriers according to family history (early criteria) or tested positive for a BRCA1 or BRCA2 mutation (later criteria)	Ovaries: multiple sections through the short axis; fallopian tubes: 40/60 had representative sections only, the other 20 were completely submitted with transverse sections	5 (8.3%) cases showed occult carcinoma; 4/5 located in fallopian tube
Leeper et al. 2002 [17]	Cross-sectional	30 BPSO specimens from women (mean age 46, range 30–65) at high risk of ovarian cancer according to family or personal history	Ovaries and tubes: first 7 cases had representative sections only, remaining 23 cases were serially sectioned	5 (16.7%) cases showed occult carcinoma; 3 located in fallopian tube
Olivier et al. 2004 [18]	Cross-sectional	38 BPO specimens and 90 BPSO specimens from women (mean age 46, range 26–74) with known BRCA1 or BRCA2 mutations or personal history of breast cancer and family history suggestive of hereditary breast and ovarian cancer	Ovaries from all procedures and tubes from BPSO procedures: sectioned in their entirety	5 (5.6%) cases from BPSO group showed occult carcinoma (all in BRCA1 carriers); 2 restricted to the fallopian tube; no occult cancers in ovary-only specimens
Lamb et al. 2006 [22]	Cross-sectional	113 BPSO specimens from women (median age 47, range 30–70) at high risk for ovarian cancer based on GOG criteria	Ovaries and tubes: sectioned at 2- to 3-mm intervals	7 (6.2%) cases showed ovarian, fallopian tube or peritoneal neoplasia; 5 were early high-grade serous tubal neoplasia
Finch et al. 2006 [21]	Cross-sectional	159 BPSO specimens from BRCA1 or BRCA2 mutation carriers (mean age 47.7, range 34–71)	Ovaries: serially sectioned; fallopian tubes initially representative sections—partway through protocol amended to submit tubes in entirety	7 (4.4%) cases showed occult carcinoma; 6 involved fallopian tube
Medeiros et al. 2006 [25]	Case-control	13 BPSO specimens from BRCA1 or BRCA2 mutation carriers (mean age 50, range 39–76) and 13 controls (mean age 58, range 43–76) undergoing surgery for benign reasons	Ovaries and tubes: sectioned at 2- to 3-mm intervals; fimbriae in some cases serially sectioned, in others by SEE-FIM protocol	5 (38%) cases showed early cancers; all in the fallopian tube; 4/5 stained positive for both p53 and MIB-1, the 5th scored positive for MIB-1 only; no cancers were found in controls
Hermsen et al. 2006 [24]	Case-control	85 BPSO specimens from high-risk women according to family history or BRCA1 or BRCA2 mutation status (median age 48, range 33–64) and 72 controls undergoing surgery for benign reasons (median age 37, range 23–79)	Sectioning details not specified	1 case of tubal carcinoma + 2 cases of severe tubal dysplasia/in situ carcinoma (3.5%) were identified in the BPSO group; no cancers were found in controls
Callahan et al. 2007 [23]	Cross-sectional	122 BPSO specimens from women with BRCA1 or BRCA2 mutations or variants (median age 46.5, range 23–76)	Ovaries and tubes: sectioned at 2- to 3-mm intervals for all cases; SEE-FIM protocol performed in a subset	7 (5.7%) cases showed occult tubal carcinoma
Finch et al. 2006 [13]	Cross-sectional	490 BPSO specimens from women with BRCA1 or BRCA2 mutations (mean age 47.6, range 19–76)	All reported cancers confirmed by review of medical records and/or pathology reports. The pathology reports were reviewed in order to correctly assign the diagnosis of ovarian, fallopian tube, or primary peritoneal cancer.	11 (2.2%) specimens showed occult cancer; 7 were identified as ovarian; 3 were classified as tubal; 1 case had positive peritoneal washings with no source of cancer identified
Powell et al. 2005 [19]	Cross-sectional	67 BPSO specimens from women with BRCA1 or BRCA2 mutations (mean age 47, range 31–64)	Ovaries and tubes: serially sectioned at 2 mm intervals (“full” adherence to specified protocol) in 20 cases; partial adherence in 21 cases; standard procedures (nonadherence) in 26 cases	7 (17%) specimens that were processed by fully or partially adherent protocols (*n* = 41) showed occult cancer; 4 were tubal and 3 were ovarian

BPO: bilateral prophylactic oophorectomy; BPSO: bilateral prophylactic salpingo-oophorectomy; GOG: gynecologic oncology group; SEE-FIM: sectioning and extensively examining the fimbriae.

**Table 2:** Serous tubal intraepithelial carcinoma in pelvic serous carcinoma cases.

Author	Study design	Study population	Sectioning Protocol	Findings
Salvador et al. 2008 [28]	Cross-sectional	16 cases of epithelial ovarian malignancy with tubes submitted in toto	fallopian tubes submitted in toto and serially sectioned every 3-4 mm	10 of the 12 cases of high-grade serous carcinoma showed either unilateral tubal mucosal involvement by TIC (*n* = 7) or tubal obliteration ipsilateral to the dominant ovarian mass (*n* = 3). In 3 of 5 selected high grade serous carcinoma cases with TICs, FISH analysis showed similar copy number changes in foci of the ovarian and fallopian tube mucosal carcinoma; one case was not synchronous and the 5th was indeterminate
Kindelberger et al. 2007 [27]	Cross-sectional	55 cases containing pelvic serous carcinoma (mean age 61.5, range 43–82)	SEE-FIM for all cases	41(75%) showed involvement of endosalpinx; 11 were classified as tubal or peritoneal primary; (9 of these had TICs); 20/30 cases classified as ovarian had TICs; 93% of TICs involved the fimbria. Of 5 ovarian cases with TICs, p53 DNA analysis showed identical mutations in at least one focus of TIC and ovarian cancer
Carlson et al. 2008 [29]	Cross-sectional	45 cases of primary peritoneal serous carcinoma in which there was either nonuniform sampling of the fallopian tube (*n* = 26) or SEE-FIM protocol (*n* = 19)	nonuniform sampling (portion of tube submitted), or SEE-FIM protocol	9 (35%) of first sampling group and 9 (47%) of second samping group showed STICs. 5/5 cases tested showed identical p53 mutations in the peritoneal and tubal lesions

FISH: fluorescence in situ hybridization; SEE-FIM: sectioning and extensively examining the fimbriae; STIC: serous tubal intraepithelial carcinoma; TIC: tubal intraepithelial carcinoma.
